# The effects of the Otago Exercise Programme on actual and perceived balance in older adults: A meta-analysis

**DOI:** 10.1371/journal.pone.0255780

**Published:** 2021-08-06

**Authors:** Huei-Ling Chiu, Ting-Ting Yeh, Yun-Ting Lo, Pei-Jung Liang, Shu-Chun Lee

**Affiliations:** 1 School of Gerontology Health Management, College of Nursing, Taipei Medical University, Taipei, Taiwan; 2 Master Degree Program in Healthcare Industry, Chang Gung University, Taoyuan, Taiwan; 3 Department of Rehabilitation Medicine, Taipei Tzu Chi Hospital, Buddhist Tzu Chi Medical Foundation, New Taipei City, Taiwan; University of Malaya, MALAYSIA

## Abstract

**Objective:**

Falls are serious issues in older populations. Balance problems are a major cause of falls and may lead to fear of falling and decreased balance confidence. The Otago Exercise Programme (OEP) is an effective fall prevention program that benefits balance function and fear of falling. The primary aim of the meta-analysis was to investigate the effectiveness of the OEP intervention on actual balance performance (i.e., static, dynamic, proactive or reactive balance) and perceived balance ability (i.e., balance confidence or fear of falling) for older adults; the secondary aim was to examine which OEP protocol most improves balance in older adults.

**Methods:**

A systematic electronic review search was performed in accordance with the Preferred Reporting Items for Systematic Reviews and Meta-analysis guidelines to identify randomized controlled trials (RCTs) investigating the effects of the OEP on actual balance performance and perceived balance ability in healthy older adults, and examining which OEP training protocol and intervention format most improves balance.

**Results:**

A total of 12 RCTs were included in the analyses. The OEP exerted significant effects on static balance (Hedges’s g = 0.388; 95% confidence interval [CI] = 0.131 to 0.645), dynamic balance (g = -0.228; 95% CI = -0.352 to -0.1.4), proactive balance (g = 0.239; 95% CI = 0.061 to 0.416) and perceived balance (g = -0.184; 95% CI = -0.320 to -0.048) in older adults. Subgroup analysis indicated that the group format for the OEP was more effective for improving static (*p* = 0.008), dynamic (*p* = 0.004) and perceived balance (*p* = 0.004) than was the individual format. Sessions of >30 minutes were more effective in improving static (*p* = 0.007) and perceived balance (*p* = 0.014) than were sessions of ≤30 minutes. However, the effects of the OEP on balance were unrelated to the types of control group, training frequency and training period.

**Discussion:**

The OEP is helpful for improving actual balance including static, dynamic, and proactive balance; enhancing confidence in balance control; and reducing fear of falling in older adults. In particular, administrating the OEP in a group setting in >30-minute sessions may be the most appropriate and effective exercise protocol for improving balance.

## Introduction

Falls are a major public health issue among older adults worldwide. More than a one-third of the community-dwelling older adults and half of older adults living in the institutions fall each year [1]. Furthermore, approximately half of those who fall do so repeatedly [2]. Although not all the falls are life threatening, it has been reported that 10%–20% of falls result in severe injuries, such as fractures, that can lead to increased morbidity and decreased quality of life [3].

Balance is defined as the ability to maintain the projection of the body’s center of mass within the limits of the base of support, as in the sitting or standing position, or in transit to establish a new base of support, as during walking [4]. Balance problems are a major cause of falls and have been shown to be associated with increased fear of falling and decreased balance confidence [5,6]. Both fear of falling and balance confidence are psychological factors that are related to balance impairment and falling and result in less social participation, greater dependence in activities of daily living, and further restriction of activity [7,8]. Older adults with postural instability and concomitant fear of falling have the greater risk of falls [9].

Recent evidence has suggested that a multi-component exercise regimen focusing on flexibility, strength, balance and endurance can effectively improve balance, mobility, and physical performance as well as reduce the incidence of falls and falls-related injuries in community-dwelling older adults [10–12]. The Otago exercise programme (OEP) encompasses all the aforementioned aspects and was developed for community-dwelling older adults aged >65 years old. The OEP consists of a set of exercises for leg muscles strengthening and balance retraining exercises and is designed to prevent falls, particularly for individuals aged >80 years who have fallen in the previous year [13]. Most studies have reported the OEP to be an effective fall prevention strategy that benefits balance function and lessens fear of falling [14–17].

In their systematic review, Martin and colleagues [18] investigated the effects of a modified OEP involving a new set of exercises to improving balance in older adults and reported improvements in balance performance and functional ability. Another meta-analysis conducted a decade ago examined the effects of the OEP on mortality and falls, and the findings indicated significant reductions in the rates of mortality and falls over a 12-month period [19]. Improving balance can reduce the likelihood of falls, but no meta-analysis has investigated the effectiveness of the OEP on actual balance performance including static, dynamic, proactive, and reactive balance. Balance is highly task-specific so specific balance categories should be evaluated separately. Moreover, the most appropriate and effective exercise training protocol (minutes per session, session frequency, and total intervention period) and intervention format (group or individual) remains unclear. Therefore, the primary purpose of this systematic review and meta-analysis was to investigate the effects of the OEP intervention on actual balance performance (e.g., static, dynamic, proactive, and reactive balance) and perceived balance ability (e.g., balance confidence and fear of falling) in older adults; the secondary purpose was to investigate which OEP protocol can most greatly improve balance improvement in older adults. Establishing an evidence-based intervention that can reduce fall, increase balance performance and perceived balance ability in older adults is of high importance.

## Methods

### Reporting standards

Systematic identification of the published literature was performed according to Preferred Reporting Items for Systematic Reviews and Meta-analysis (PRISMA) guidelines [20].

### Search strategy

A search for relevant studies was performed using the Physiotherapy Evidence Database (PEDro), PubMed, Embase, Web of Science, Oxford, Medline, and CINAHL databases from inception until 26 February 2021 by using the following keywords: “old” OR “aged” OR “elderly” AND “Otago exercise”. databases.

### Selection criteria

Selection criteria were established on the basis of the Population Intervention Comparison Outcomes (PICO) framework [21] as follows: 1) Population: the population must be older adults aged >65 years without any neurological diseases; 2) Intervention: the intervention must comprise the original OEP with either an individual or group format; 3) Comparison: the comparison group must be alternative active treatment methods or no treatment; 4) Outcome: the reported outcomes must include actual and perceived balance-related parameters. In addition to the aforementioned PICO components, studies must be published in English articles, employ a randomized controlled trials (RCTs) design, and provide sufficient statistical data for effect size calculation. Study protocols and review articles, studies including older adults with neurological diseases, and studies with low quality based on a PEDro scale score <3 were excluded.

### Data extraction

Two independent authors (SCL and YTL) extracted the following data from all selected RCTs: participants details (characteristics, sample size, age, and sex), intervention characteristics (format and frequency), outcomes of interest and results. If the two authors held different opinions, then the third author (HLC) resolved any disputes.

### Outcome measures

Balance is highly task-specific; therefore, specific balance categories should be evaluated separately. The model proposed by Shumway-Cook and Woollacott divides balance performance into four components including static balance, dynamic balance, proactive balance, and reactive balance [22]. Several studies have indicated that there are only weak to moderate correlations among the four types of balance in older adults [23,24] Therefore, this meta-analysis focused separately on these balance outcome categories.

Static balance is the ability to maintain postural stability and orientation with center of mass over the base of support and body at rest. It can be evaluated with tasks that require standing on one or both legs standing in a stationary posture. The time required to maintain balance and the postural sway measured using a force plate are considered proxies for static balance [25,26]. Dynamic balance is defined as the ability to maintain stability during weight shifting, often while changing the base of support. The Timed Up and Go (TUG) test examines gait speed, stride length, and balance in locomotion. Thus, the TUG can be used to examine dynamic balance [27]. Proactive balance involves postural adjustment during a self-initiated movement, and the Functional Reach Test (FRT) is the preferred proxy [28]. Reactive balance is balance control in response to an unexpected perturbation [29], but the current meta-analysis could not include reactive balance because the output measures in the existing research are limited. A previous study reported a strong relationship between perceived balance and actual balance performance [30]. Perceived balance is often measured using self-reported questionnaires, such as the Falls Efficacy Scale (FES) [31], which assesses fear of falling in performing daily activities, and CONFbal scale, which measures an older adult’s confidence in maintaining balance [32]. If a study used other tests other than those mentioned, we included in our quantitative analyses those tests that were the most similar to the aforementioned tests in terms of their temporal and spatial structure.

### Assessment of methodological quality

Two independent authors (SCL and YTL) independently applied the selection criteria and assessed the methodological quality of the studies by using the PEDro scale [33]. A total PEDro score is determined by counting the number of criteria that are satisfied, except that scale item 1 is not used to generate the total score; thus the score is calculated out of a total score of 10. According to the previous research and definition of PEDro scale [33,34], the score of 6–10, 4 or 5, and <3 indicates high, moderate, and low study quality. Low-quality studies were excluded from this analysis.

### Data analysis

We performed the meta-analysis by using Comprehensive Meta-Analysis (VMA) Version 2.0 (Biosta, Inc. USA). The effect size was estimated using Hedges’s g coefficient and 95% confidence intervals (CIs). The magnitude of Hedges’ g was interpreted using Cohen’s convention of 0.2, 0.5, and 0.8 respectively indicating small, moderate, and large effects [35]. Heterogeneity was estimated using the *Q* statistic and *I*^2^ test. Each quartile was considered a heterogeneity interval, and values of 0%–25%, >25%–50%, >50%–75%, and >75% indicated no, minor, moderate, and high heterogeneity, respectively [36]. All overall effect size values in this study were examined using a fixed-effects model. Furthermore, subgroup analysis was further employed to determine whether the characteristics of the intervention or the types of control group affected the effect size among studies. In addition, Egger’s regression intercept was used to assess publication bias.

## Results

### Selection and characteristics of studies

**Fig 1:**
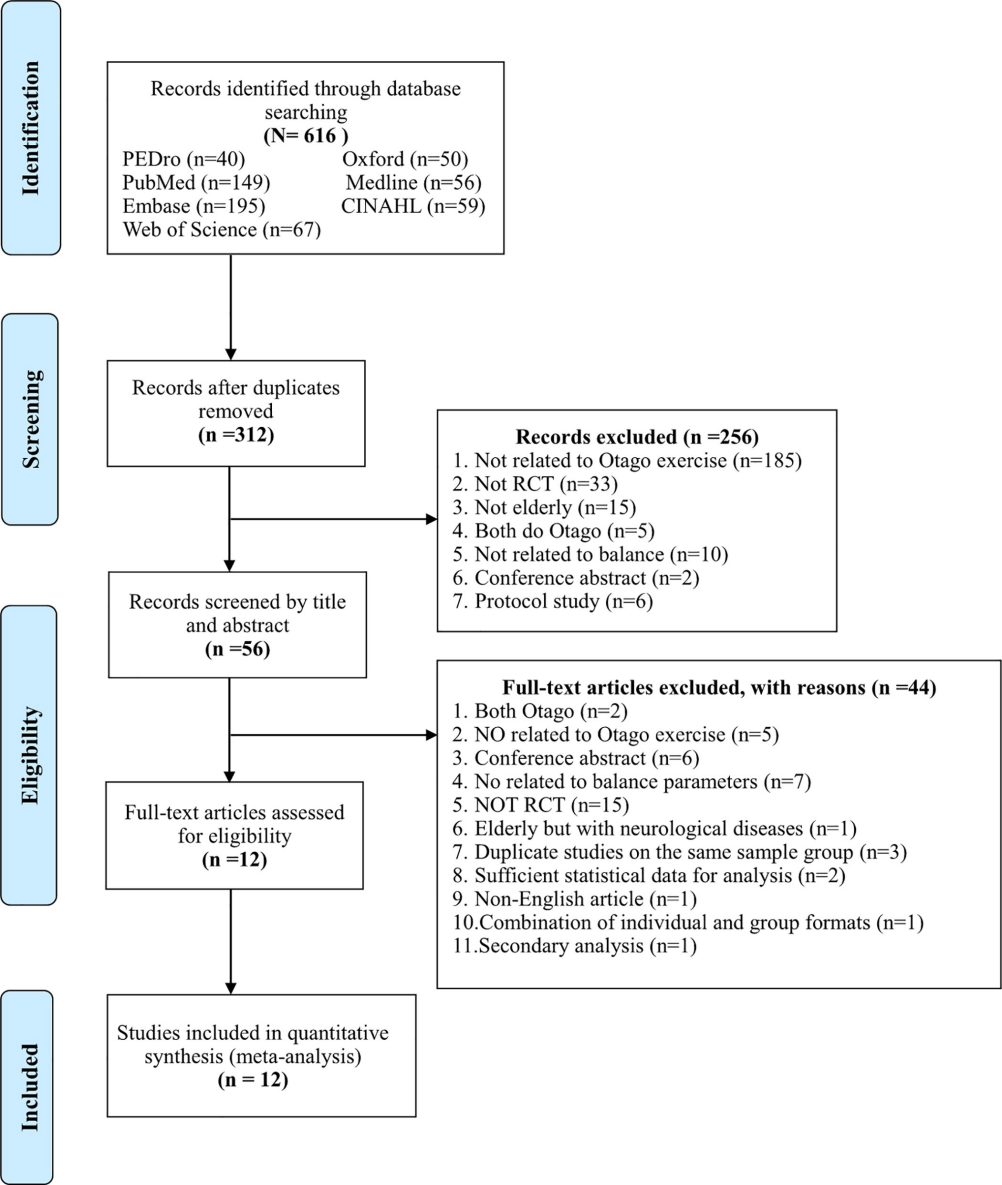
PRISMA flow diagram of the study selection process (RCT, randomized controlled trial).

A total of 616 unique titles and abstracts were retrieved from the literature research (Fig 1). Twelve RCTs published in peer-reviewed journals met the selection criteria [15–17,37–45]. Among these 12 RCTs, 4, 9, 4, and 5 analyzed the effects of the OEP on static [17,38,41,44], dynamic [15,17,37–40,42–44], proactive [40,43–45], and perceived balance [16,37,40–42], respectively.

The 12 RCTs comprised a total of 2807 participants, with sample sizes ranging from 10 to 125. The mean age of participants was 76.34±4.84 years old. Five studies applied the OEP for older adults who had experienced falls [17,39–40,43,45] and indicated that the OEP was safe and feasible for even those older individuals prone to falls. Eight studies (of 12) administrated the OEP as an individual program [16–17,37,39–41,43,45]; however, four studies administrated the OEP within a group [15,38,42,44]. Participants in active control groups participating in other exercise programs, such as Tai Chi [44] or Yoga [41]. Control group receiving no treatment maintained regular activities [15–17,37–40,42,43,45]. The training session length ranged from 20 to 60 minutes, the frequency was 2 or 3 sessions per week, and the intervention lasted 12–52 weeks, representing 1080–4320 minutes in total. The most common training protocol was 30 minutes per session for 3 sessions per week for 12 or 24 weeks, totaling 2160 minutes. The compliance rates were 77–93 (85±11) %, defined as the frequency of attendance to exercise sessions [15,38], and 24–67 (33±13) %, reported on the number of participants who adhered to the exercise protocol [17,37,40,43,45]. Compliance was not reported in 5 studies [16,39,41,42,44]. Table 1 lists the characteristics of the included studies.

**Table 1:** Characteristics of included studies.

Study	Participants	Intervention characterization	Outcome of interests/Results
Arkkukangas et al., 2019 [37]	**Characteristics:** Older adults **Total N:** EG1/EG2/CG: 61/58/56 **Complete N:** EG1/EG2/CG: 54/52/55 **Age (yrs):** EG1/EG2/CG: 83.0±5.0/84±4.1/82.0±4.7 **Gender (Female%):** EG1/EG2/CG: 67/69/73	**EG1**: Otago exercise **EG2**: Otago exercise plus motivational interviewing **CG**: Regular care **Format**: Individual **Frequency**: Minutes per session: 30 Session per week: 3 Total weeks: 12	**Dynamic balance** Mini-BEST test (score) **Perceived balance** FES (score)
Benavent-Caballer et al., 2016 [38]	**Characteristics:** Older adults **Total N:** EG/CG: 28/23 **Complete N:** EG/CG: 28/23 **Age (yrs):** EG/CG: 69.1±4.0/69.0±3.3 **Gender (Female%):** EG/CG: 82/69	**EG**: Otago exercise **CG**: No intervention **Format**: Group **Frequency**: Minutes per session: 45 Session per week: 3 Total weeks: 16	**Static balance** One leg stance test (s) **Dynamic balance** BBS (score) TUG (s)
Dadgari et al., 2016 [39]	**Characteristics:** Older fallers **Total N:** EG/CG: 160/157 **Complete N:** EG/CG: 160/157 **Age (yrs):** EG/CG: 70.60±5.80/70.06±5.20	**EG**: Otago exercise **CG**: Regular care **Format**: Individual **Frequency**: Minutes per session: 60 Session per week: 3 Total weeks: 24	**Dynamic balance** TUG (s) BBS (score)
Elley et al., 2008 [40]	**Characteristics:** Older fallers **Total N:** EG/CG: 155/157 **Complete N:** EG/CG: 135/145 **Age (yrs):** EG/CG: 80.4±4.8/81.1±5.3 **Gender (Female%):** EG/CG: 68/70	**EG**: Otago exercise **CG**: Regular care **Format**: Individual **Frequency**: Total weeks: 52	**Dynamic balance** TUG (s) **Proactive balance** Step test (n) **Perceived balance** mFES (%)
Iliffe et al., 2015 [16]	**Characteristics:** Older adults **Total N:** EG/CG1/CG2: 410/387/457 **Complete N:** CONFbal: EG/CG1/CG2:179/183/218 FES-I: EG/CG1/CG2:185/188/220 **Age (yrs):** EG/CG1/CG2:72.8±5.8/72.9±6.1/73.1±6.2 **Gender (Female%):** EG/CG1/CG2: 63/62/62	**EG**: Otago exercise **CG1**: Falls Management Exercise Programme **CG2**: Regular care **Format**: Individual **Frequency**: EG: Minutes per session: 30 Session per week: 3 Total weeks: 24 CG1: Minutes per session: 60 Session per week: 1 Total weeks: 24	**Perceived balance** CONFbal (score) FES-I (score)
Kocic et al., 2018 [15]	**Characteristics:** Older adults **Total N:** EG/CG: 38/39 **Complete N:** EG/CG: 27/33 **Age (yrs):** EG/CG: 78.3±8.1/78.5±7.2 **Gender (Female%):** EG/CG: 74/59	**EG**: Otago exercise **CG**: Regular care **Format**: Group **Frequency**: Minutes per session: 40 Session per week: 3 Total weeks: 24	**Dynamic balance** BBS (score) TUG (s)
Lee et al., 2017 [41]	**Characteristics:** Older females **Total N:** EG1/EG2/CG: 10/10/10 **Complete N:** EG1/EG2/CG: 10/10/10 **Age (yrs):** EG1/EG2/CG: 72.60±2.67/76.40±5.54/75.80±5.47 **Gender (Female%):** EG1/EG2/CG: 100/100/100	**EG1**: Augmented reality Otago exercise **EG2**: Self-Otago exercise **CG**: Yoga **Format**: Individual **Frequency**: Minutes per session: 60 Session per week: 3 Total weeks: 12	**Static balance** Postural sway (cm)-HoE Postural sway (cm)-SD-x Postural sway (cm)-EO CoP-x Postural sway (cm)-EC CoP-x **Perceived balance** Morse Fall Scale (score)
Leem et al., 2019 [42]	**Characteristics:** Older females **Total N:** EG1/EG2/CG: 10/10/10 **Complete N:** EG1/EG2/CG: 10/10/10 **Age (yrs):** EG1/EG2/CG:79.50±4.55/76.30±5.16/81.10±3.07 **Gender (Female%):** EG1/EG2/CG: 100/100/100	**EG1**: Otago exercise plus action observation **EG2**: Otago exercise **CG**: Regular care **Format**: Group **Frequency**: Minutes per session: 50 Session per week: 3 Total weeks: 12	**Dynamic balance** TUG (s) **Perceived balance** FES (score)
Liew et al., 2019 [43]	**Characteristics:** Older fallers **Total N:** EG/CG: 34/33 **Complete N:** EG/CG: 24/24 **Age (yrs):** EG/CG: 75.9±7.0/75.2±7.2 **Gender (Female%):** EG/CG: 75/83	**EG**: Otago exercise (Using ankle punching bag) **CG**: Regular care **Format**: Individual **Frequency**: Minutes per session: 30 Session per week: 3 Total weeks: 12	**Dynamic balance** TUG (s) **Proactive balance** Functional reach test (cm)
Liu-Ambrose et al., 2008 [17]	**Characteristics:** Older fallers **Total N:** EG/CG: 31/28 **Complete N:** Postural sway: EG/CG: 27/23 TUG: EG/CG: 28/24 **Age (yrs):** EG/CG: 81.4±6.2/83.1±6.3 **Gender (Female%):** EG/CG: 71/68	**EG**: Otago exercise **CG**: Regular care **Format**: Individual **Frequency**: Minutes per session: 30 Session per week: 3 Total weeks: 24	**Static balance** Postural sway (mm) **Dynamic balance** TUG (s)
Son et al., 2016 [44]	**Characteristics:** Older Females **Total N:** EG/CG: 24/26 **Complete N:** EG/CG: 24/21 **Age (yrs):** EG/CG: 71.5±3.6/72.8±4.7 **Gender (Female%):** EG/CG: 100/100	**EG**: Otago exercise **CG**: Tai Chi **Format**: Group **Frequency**: Minutes per session: 60 Session per week: 2 Total weeks: 12	**Static balance** One leg stance test (s) **Dynamic balance** TUG (s) **Proactive balance** Functional reach test (cm)
Yang et al., 2012[45]	**Characteristics:** Older fallers **Total N:** EG/CG: 82/83 **Complete N:** EG/CG: 59/62 **Age (yrs):** EG/CG: 81.0±5.9/80.1±6.4 **Gender (Female%):** EG/CG: 45/43	**EG**: Otago exercise **CG**: Regular care **Format**: Individual **Frequency**: Minutes per session: 20 Session per week: 5 Total weeks: 24	**Proactive balance** Functional reach test (cm) Step test (n)

BBS: Berg Balance Scale; CG: Control Group; CONFbal: Confidence in Balance; CoP: Center of Pressure; EC: Eyes Closed; EG: Experimental Group; EO: Eyes Closed; FES: Falls Efficacy Scale; FES-I: Falls Efficacy Scale International; HoE: Height of Ellipse; mFES: Modified Falls Efficacy Scale; Mini-BEST test: Mini Balance Evaluation Systems Test; SD: Standard Deviation; TUG: Timed Up and Go Test.

### Quality assessment

The average PEDro scale score of the 12 RCTs was 6.6 out of 10, suggesting the study designs were of high quality, with only one study [16] determined to have fair quality (Table 2). The included studies presented a reasonable control over eligibility criteria, random allocation, baseline comparability, between-group comparisons, and point estimates. However, a major design limitation was the lack of blinding procedures; no study performed blinding of both participants and therapists.

**Table 2:** Physiotherapy Evidence Database (PEDro) scale scores of the reviews.

Study	1	2	3	4	5	6	7	8	9	10	11	Total Score	Quality
Arkkukangas et al., 2019 [37]	O	O	X	O	X	X	O	O	O	O	O	7/10	High
Benavent-Caballer et al., 2016 [38]	O	O	O	O	X	X	O	X	O	O	O	7/10	High
Dadgari et al., 2016 [39]	O	O	X	O	O	X	O	O	O	O	O	8/10	High
Elley et al., 2008 [40]	O	O	O	O	X	X	O	O	X	O	O	7/10	High
Iliffe et al., 2015 [16]	O	O	X	O	X	X	X	X	X	O	O	4/10	Fair
Kocic et al., 2018 [15]	O	O	X	O	X	X	O	O	X	O	O	6/10	High
Lee et al., 2017 [41]	O	O	X	O	X	X	X	O	O	O	O	6/10	High
Leem et al., 2019 [42]	O	O	X	O	X	X	X	O	O	O	O	6/10	High
Liew et al., 2018 [43]	O	O	O	O	X	X	O	X	X	O	O	6/10	High
Liu-Ambrose et al., 2008 [17]	O	O	O	O	X	X	O	X	O	O	O	7/10	High
Son et al., 2016 [44]	O	O	O	O	X	X	O	O	O	O	O	8/10	High
Yang et al., 2012 [45]	O	O	O	O	X	X	O	X	O	O	O	7/10	High

1: Eligibility criteria 2: Random allocation 3: Concealed allocation 4: Baseline comparability 5: Blind subjects 6: Blind therapists 7: Blind assessors 8: Adequate follow-up 9: Intention-to-treat analysis 10: Between-group comparisons 11: Point estimates.

### Effects on static balance, dynamic balance, proactive balance, and perceived balance

The effect of OEP on static balance in older adults demonstrated a significant but small effect (Hedges’s g = 0.388, 95% CI 0.131 to 0.645, p = 0.003) by four studies [17,38,41,44]. The results of heterogeneity testing revealed a high level of heterogeneity (Q = 14.17, *p* = 0.003, *I*^*2*^ = 78.83%). The results of Egger’s regression intercept indicated that publication bias was not significant (*p =* 0.732).

The effect of OEP on dynamic balance in older adults demonstrated a significant but small effect (Hedges’s g = -0.228, the 95% CI -0.352 to -0.104, p<0.001) by nine studies [15,17,37–40,42–44]. The results of heterogeneity testing showed a moderate level of heterogeneity (Q = 16.30, *p* = 0.0380, *I*^*2*^ = 50.925%). The results of Egger’s regression intercept test indicated that publication bias was not significant (*p =* 0.100).

The effect of OEP on proactive balance in older adults demonstrated a significant but small effect (Hedges’s g = 0.239, 95% CI 0.061 to 0.416, p = 0.009) by four RCTs [40,43–45]. The results of heterogeneity testing revealed a high level of heterogeneity (Q = 20.49, *p* = 0.000, *I*^*2*^ = 85.36%). The results of Egger’s regression intercept test indicated that publication bias was not significant (*p = *0.264).

**Fig 2:**
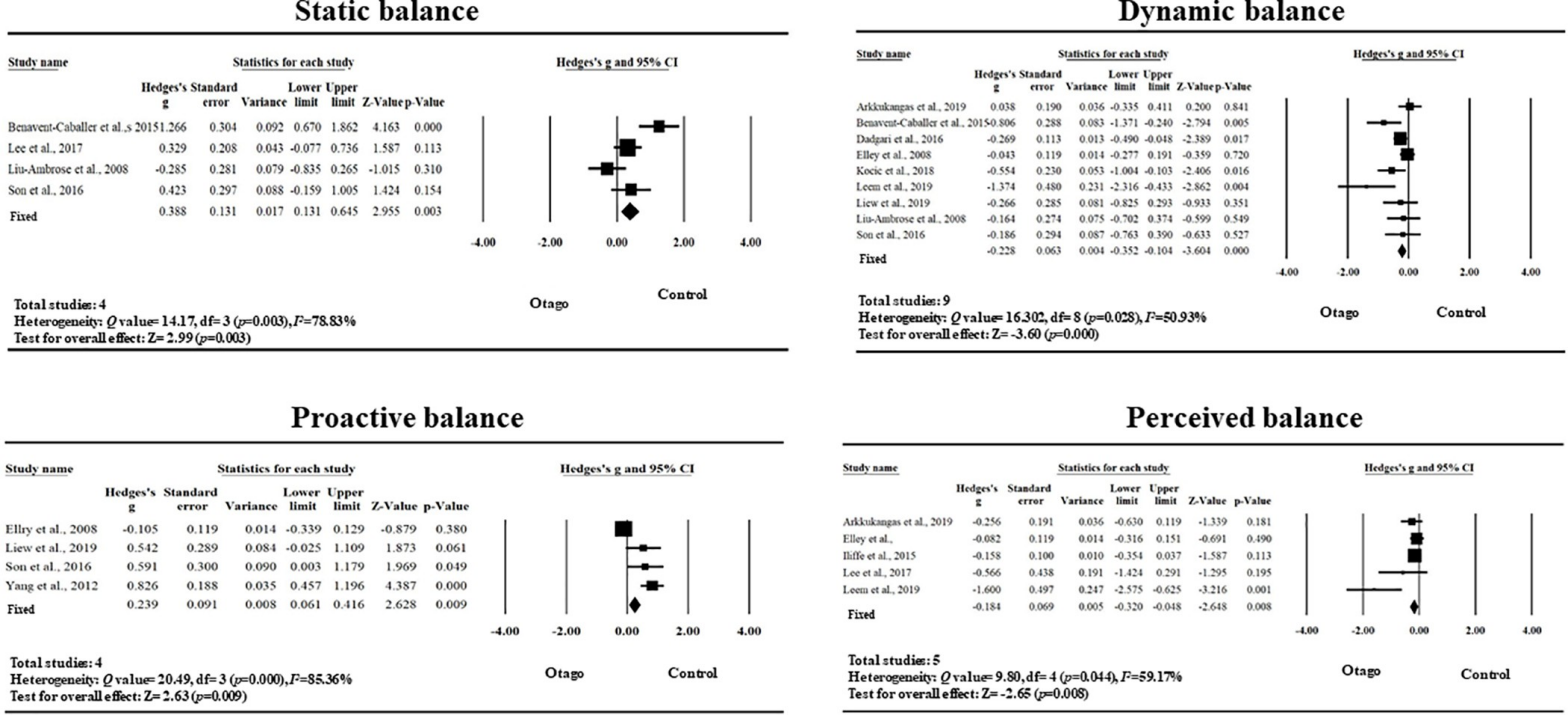
Effects of the OEP on static balance, dynamic balance, proactive balance and perceived balance (OEP, Otago Exercise Programme).

The effect of OEP on perceived balance demonstrated a significant but small effect (Hedges’ g = -0.184, 95% CI = -0.320 to -0.048, p = 0.008) by five studies [16,37,40–42]. Heterogeneity testing revealed a moderate level of heterogeneity among the studies (Q = 9.796, *p* = 0.044, *I*^2^ = 59.166%). The results of Egger’s regression intercept test indicated that publication bias was not significant (*p =* 0.062; Fig 2).

Therefore, the effect of OEP exerted significant effects on static balance, dynamic balance, proactive balance and perceived balance in older adults.

### Subgroup analysis

Because the results revealed that the OEP exerted significant effects on static, dynamic, proactive, and perceived balance, we performed subgroup analysis to determine whether the characteristics of the intervention or the types of control group influenced the effect size. Group OEP interventions were discovered to exert a greater effect on static (p = 0.008), dynamic (p = 0.004) and perceived balance (p = 0.004) than did individual interventions. Sessions of >30 minutes were more effective than those of ≤30 minutes on static (p = 0.007) and perceived balance (p = 0.014). However, the effects of the OEP on balance did not differ by control group type, training frequency, or training period (Table 3).

**Table 3:** Subgroup analysis of study participants and intervention characteristics.

	Static balance				Dynamic balance				Proactive balance				Perceived balance			
Variables	K	Hedges’s g (95%CI)	*p*^a^	*p*^b^	K	Hedges’s g (95%CI)	*p*^a^	*p*^b^	K	Hedges’s g (95%CI)	*p*^a^	*p*^b^	K	Hedges’s g (95%CI)	*p*^a^	*p*^b^
**Intervention format**				0.008*				0.004*				0.218				0.004*
Group	2	**0.83 (0.42, 1.25)**	**<0.001**		4	**-0.60 (-0.89, -0.32)**	**<0.001**		1	0.59 (0.00, 1.18)	0.049		1	**-1.60 (-2.58, -0.63)**	**0.001**	
Individual	2	0.11 (-0.22, 0.44)	0.502		5	-0.141 (-0.28, -0.00)	0.044		3	0.20 (0.02, 0.39)	0.033		4	-0.16 (-0.29, -0.02)	0.026	
**Control group**				0.798				0.884				0.218				0.376
Active control	2	0.36 (0.03, 0.69)	0.034		1	-0.19 (-0.76, 0.39)	0.527		1	0.59 (0.00, 1.18)	0.049		1	-0.57 (-1.42, 0.29)	0.195	
No treatment	2	0.43 (0.02, 0.83)	0.038		8	-0.23 (-0.36, -0.10)	<0.001		3	0.20 (0.02, 0.39)	0.033		4	-0.17 (-0.31, -0.04)	0.013	
**Minutes per session**				0.007*				0.057				0.657				0.014*
>30min	3	**0.58 (0.29, 0.87)**	**<0.001**		5	-0.39 (-0.57, -0.22)	<0.001		1	0.59 (0.00, 1.18)	0.049		2	**-1.02 (-1.66, -0.37)**	**0.002**	
≤30min	1	-0.29 (-0.84, 0.27)	0.310		3	-0.08 (-0.35, 0.19)	0.546		2	0.74 (0.43, 1.05)	0.000		2	-0.18 (-0.35, -0.01)	0.043	
undisclosed					1				1				1			
**Session per week**				0.895				0.687				0.657				1.000
>2 times/week	3	0.38 (0.09, 0.67)	0.010		7	-0.31 (-0.46, -0.16)	<0.001		3	0.74 (0.43, 1.05)	0.000		4	-0.24 (-0.40, -0.07)	0.006	
≤2 times/week	1	0.42 (-0.16, 1.01)	0.154		1	-0.19 (-0.76, 0.39)	0.527		0	0.59 (0.00, 1.18)	0.049		0	--	--	
undisclosed					1				1				1			
**Total weeks**				0.798				0.714				0.081				0.078
>12weeks	2	0.43 (0.02, 0.83)	0.038		5	-0.24 (-0.38, -0.10)	0.001		2	0.16 (-0.04, 0.36)	0.108		2	-0.13 (-0.28, 0.02)	0.097	
≤12weeks	2	0.36 (0.03, 0.69)	0.034		4	-0.19 (-0.45, 0.08)	0.167		2	0.57 (0.16, 0.97)	0.007		3	-0.45 (-0.77, -0.12)	0.007	
**Total minutes**				0.895				0.296				0.353				0.218
>2000minutes	3	0.38 (0.09, 0.67)	0.010		4	-0.35 (-0.53, -0.18)	<0.001		2	0.83 (0.46, 1.20)	0.000		2	-0.18 (-0.37, 0.01)	0.067	
≤2000minutes	1	0.42 (-0.16, 1.01)	0.154		4	-0.19 (-0.45, 0.08)	0.167		1	0.57 (0.16, 0.97)	0.007		2	-0.43 (-0.78, -0.08)	0.016	
undisclosed					1				1				1			

^a^, within studies

^b^, between studies; CI, confidence interval

*p < 0.05.

## Discussion

Balance is a key component of many activities of older adults’ daily living, ranging from simple activities such as quiet standing to more complex activities such as walking while talking [46]. Balance improvement benefits older adults’ physical function, independence, and physical activity [47], leading to additional health benefits [48]. The primary aim of this systematic review and meta-analysis was to examine the overall effects of the OEP on older adults’ actual and perceived balance and the secondary aim was to investigate which OEP protocol could maximize balance improvement. The 12 RCTs reported more favorable outcomes for the OEP groups than for the control group. A significant and small effect was observed on static, dynamic, proactive, and perceived balance. Furthermore, subgroup analysis further indicated that the group format was more effective than the individual format for improving balance performance with the OEP. A training session duration of >30 minutes was determined to be the most appropriate and effective for improving balance. However, the effects of the OEP on balance were not related to the control group type, training frequency, or overall intervention period.

### Effects of the OEP on actual balance performance

Balance control is complex and multifactorial. Age-related physiological changes include reductions in muscle strength, joint range of motion, and reaction time as well as the deterioration of sensory systems. These changes negatively influence older adults’ balance control and may lead to various levels of balance dysfunction [49]. Multimodal exercises, such as the OEP, that focus on muscle strengthening and balance training are recommended. Exercise guidelines for older adults suggest multimodal regimens [50,51]. Studies have indicated that multimodal exercise is a comprehensive approach to fall prevention [52,53] and particularly effective for improving dynamic standing balance [54]. Dynamic balance is more crucial than other types of balance because it is a fundamental component of mobility in everyday life that allows a person to move safely and easily without difficulty and avoid falls [27].

The beneficial effects of the OEP on balance can be attributed to its resistance and balance training elements. Resistance training exercises included in the OEP improve strength in the large muscle groups of the lower limbs, including the quadriceps (e.g., front knee strengthening), hamstrings (e.g., back knee strengthening), hip abductors (e.g., side hip strengthening), calf muscles (e.g., calf raises), and tibialis anterior muscles (e.g., toe raises). These muscles are strongly correlated with balance, and their strength can be enhanced through resistance training to improve balance [55–57]. Improvement in overall balance resulting from the OEP can be attributed to its inclusion of tasks training multiple domains of balance, including static (e.g., heel-toe and one leg standing), dynamic (e.g., toe walking and sideways walking), and proactive balancing tasks (e.g., knee bends and sit to stand exercises). This reflects the principles of specificity in balance exercise; the more an exercise targets a specific motor task, the greater is the carryover from that exercise to performing real related tasks [58].

The improvement in balance through OEP intervention may be related to improvements in cognition. A study reported an association between cognitive function and balance ability in older adults [59]. This association might be due to cognitive and balance networks sharing common neuronal pathways [60]. A lower gray matter volume was reported to be associated with not only poorer cognitive function but also postural instability [61]. Aerobic exercise can improve cognition [62]; however, some studies have indicated that resistance [63] and balance training [64] may prevent or delay the age-related decline in cognitive functions. Therefore, the OEP may bolster cognitive function through exercise and balance training, further improving balance.

### Effects of the OEP on perceived balance ability

Fear of falling is defined as a lasting concern about falling that can lead individuals to avoid activities that they remain capable of performing [65]. Balance confidence is defined as individuals’ belief in their abilities to maintain balance and further exacerbate activity avoidance due to a lack of confidence in the ability to maintain balance and not fall during feared activities [66]. Both fear of falling and balance confidence are psychological problems, that reduce activity levels and impair physical functions. Impaired physical function, in turn, affects neuromuscular ability and increases the risks of falling and its associated fear, thus perpetuating a vicious cycle [31]. Fear of falling and low balance confidence are reported in both fallers and non-fallers, and can potentially be more debilitating than a fall itself [67]. Thus, fear of falling and lack of balance confidence have been identified as key components in fall prevention programs. Resistance [68] or balance training [69] or a combination of both [70] have been shown to improve balance confidence and fall self-efficacy. In line with the findings of previous studies, the results of our meta-analysis indicated that the OEP, with its resistance and balance training, can reduce the fear of falling and enhance balance confidence. Interventions to overcome the fear of falling and build balance confidence are crucial because fall-related psychological factors can lead to various adverse health outcomes [71].

### Intervention characteristics

Exercise programs, including home-based (individual-based) and group-based programs, have yielded promising results, reducing the risk of falls in older adults [72,73]. Previous studies have reported conflicting results regarding whether group or home exercise is more effective [74–76] However, each approach has its own strengths and weaknesses. The decision to prescribe either approach may be driven by factors such as access, individual preference, available resources, the need for individually tailored exercises, and levels of supervision and socialization [73]. Group-based programs allow interactions and communication between participants and therapists as well as among participants, providing psychosocial support and a sufficient dose of overall exercise [77,78]. By contrast, home-based programs provide opportunities for participants to exercise without commuting, can be followed for an unlimited time by participants, and may be easier to sustain than group-based programs [79,80]. Although the OEP was originally designed as an individually tailored home exercise program, our subgroup analysis indicated that a group-based OEP was more effective than a home-based OEP for improving static, dynamic, and perceived balance. A recent meta-analysis suggested that a combination of a supervised group exercises and self-directed home-based exercises may be optimal for improving functional performance and preventing injurious falls in community-dwelling older adults with a risk of falling [81].

Our subgroup analysis revealed that >30-minute OEP training sessions were the most effective for improving balance, but the effects of the OEP on balance were not found to be related to the training period (total training week or total training minutes) or frequency (session per week). In fact, the American College of Sports Medicine (ACSM) guidelines for older adults recommend that all healthy older adults perform regular exercise for a minimum of 30 minutes and five days per week [82]. The OEP manual also suggests a training session duration of 30 minutes, with three training sessions each week and go for a walk at least twice every week for 6 months; although a broad range of intervention periods (12–52 weeks), training frequencies (2 or 3 sessions per week), session durations (20–60 minutes), and total OEP intervention times (1080–4320 minutes in total) was employed in the included studies [15–17,37–45].

### Strengths and limitations

Our systematic review and meta-analysis has several strengths. First, the literature search was performed in accordance with the PRISMA guidelines. An electronic search of major databases was conducted using keywords based on the clinical recommendations of the PICO model. Second, the risk of bias was assessed using the PEDro scale, enabling a comprehensive and detailed analysis of the methodological quality and the effect of bias on the treatment outcomes [83]. In this literature review, 12 high-quality RCTs were included that provided stronger evidence than provided by studies with other designs.

This study has some limitations that should be addressed. The heterogeneity between studies was considerable and may be due to the different intervention characteristics or training formats. Therefore, subgroup analysis was performed. Although the overall quality of included studies was high, all of them failed to impose complete blinding procedures, and this might limit the credibility of our results. Future RCTs should be blind as many parties as possible in the trial to minimize bias and maximize the validity of the results. Moreover, independent therapists and outcome assessors should be used, and participants should be blinded to their group allocation by including them in a waitlist control group that receives intervention at some later point or an active control group that receives an alternative intervention. Additional high-quality RCTs should examine the benefits of the OEP and particularly focus on implementing blinding procedures are needed.

The model proposed by Shumway-Cook and Woollacott divided balance capacity into four components: static, dynamic, proactive and reactive balance [22]. However, the current meta-analysis could not include reactive balance because of limited output measures. The lack of output measures may be related to the OEP not including reactive balance training, such as perturbation training. Reactive balance is crucial for restoring balance in response to an unexpected perturbation. An inadequate reaction to an unexpected disturbance can result in falls because the assessments performed in the included studies were mostly concerned with balance control under self-initiated conditions, future studies should examine the effects of the OEP on reactive balance in older adults [84]. The OEP supplemented with reactive balance training could possibly improve comprehensive balance capacity including static, dynamic, proactive and reactive balance.

Another limitation may be that the intervention period was not as recommended by the OEP manual (i.e., 24 weeks). Therefore, we performed subgroup analysis by investigating the effects of heterogeneous training frequencies. An optimal training duration of >30 minutes per session was identified, consistent with the original suggestions of the OEP manual. Another concern is the fact that the outcome measures of the included articles were mostly based on clinical tests. Clinical tests are commonly used because they are cost effective, time efficient, and easy to administer. Improvements in clinical test results may suggest clinically meaningful changes. However, a sophisticated laboratory instrument could not only provide an objective assessments of balance control under various conditions but also detects subtle changes in performing that clinical tests could not. Therefore, laboratory assessments such as the use of force plates and the GAITRite system to collect information regarding the center of pressure data and gait kinematics, respectively, should be included in future studies.

### Perspective

The OEP is a multimodal exercise regimen focusing on flexibility, strength, balance, and endurance exercises designed to prevent falls. This meta-analysis of 12 high-quality RCTs provides evidence that the OEP significantly improves actual balance performance, including static, dynamic, and proactive balance, as well as perceived balance ability. In particular, administrating the OEP in a group setting in 30-minute sessions may be the most appropriate and effective protocol for improving balance. Therefore, the OEP is highly recommended for community-dwelling older adults to improve their actual balance performance and enhance their confidence in balance control.

## Conclusions

The primary purpose of this systematic review and meta-analysis was to investigate the effects of OEP interventions on actual balance performance (i.e., static, dynamic, proactive or reactive balance) and perceived balance (i.e., balance confidence or fear of falling) in older adults; the secondary purpose was to investigate which OEP protocol leads to the greatest balance improvements. This meta-analysis provides evidence that the OEP is helpful for improving actual static, dynamic, and proactive balance; enhancing confidence in balance control; and reducing the fear of falling. In particular, administrating the OEP in a group setting in 30-minute sessions may be the most appropriate and effective protocol for improving balance. Future studies should examine the effects of the OEP on reactive balance performance, adopt a high-quality study design with blinding procedures, and finally utilize laboratory outcome measurements for precise assessment.

## Supporting information

S1 Appendix PRISMA 2009 checklist. (DOC)
